# Comparison of Inter-Rater and Intra-Rater Reliability of Raters with Different Levels of Experience When Using Landing Error Scoring System (LESS) in Field-Based Screening of Professional Football Players

**DOI:** 10.3390/sports12090242

**Published:** 2024-09-03

**Authors:** Nikolaos I. Liveris, Charis Tsarbou, Sofia A. Xergia, Angelos Papadopoulos, Elias Tsepis

**Affiliations:** Department of Physiotherapy, School of Health Rehabilitation Sciences, University of Patras, Rio, GR26504 Patras, Greece; n.liveris@upatras.gr (N.I.L.); ctsarmpou@upatras.gr (C.T.); sxergia@upatras.gr (S.A.X.); angpap1997@gmail.com (A.P.)

**Keywords:** pre-season screening, reliability, field-based examination, landing, risk factors

## Abstract

It is essential for physical sports therapists to use reliable field-based tests to identify potential injury risk factors in athletes. The purpose of this study was to compare the inter- and intra-rater reliability of experienced and novice raters during use of the Landing Error Scoring System (LESS) in a field-based examination of professional football athletes. Thirty-seven male football athletes underwent pre-season LESS assessment. Two raters independently evaluated the recorded landing techniques at two separate intervals, two months apart, following the LESS standard protocol. Inter-and intra-rater values were calculated for the LESS total scores and individual scoring items. The overall LESS scores had excellent intra-rater reliability values for both the experienced (interclass correlation coefficient (ICC) = 0.95, 95% CI, 0.89–0.97; *p* < 0.001) and novice rater (ICC = 0.95, 95% CI, 0.90–0.97; *p* < 0.001), and very good to excellent inter-rater values for the first (ICC = 0.90, 95% CI, 0.77–0.95; *p* < 0.001) and second (ICC = 0.86, 95% CI, 0.71–0.93; *p* < 0.001) evaluation. Most of the individual scoring items ranged from moderate to perfect agreement. In conclusion, sports physical therapists, regardless of experience, can reliably use the LESS’s total score, through video analysis of the regime. Individual scoring items can inform clinicians about impairments in the landing mechanism but data should be interpreted cautiously.

## 1. Introduction

Athletes’ screening is considered an essential first step in order to identify potential risk factors that may increase the likelihood of injury. The information gained by the application of screening tests can guide sports physical therapists to apply targeted preventive interventions that may reduce the risk of injury [1,2]. Despite pre-season screening being a standard procedure for football teams, sports injuries remain a substantial problem for teams’ performance and financial integrity [3]. The multifactorial and complex nature of injuries as well as the questionable validity of many screening tests make the prevention of injuries a challenging task [4,5]. Biomechanical predisposing factors, such as kinematic and kinetic parameters of landing and cutting movements, play a key role in the injury mechanism [6,7]. Laboratory-based examination using sophisticated motion analysis systems can identify kinematic and kinetic impairments during jumping or cutting tasks [6,8]. These systems are highly reliable; however, they are costly, time-consuming, and challenging to use in a field-based setting [9]. Therefore, the availability of reliable, low-cost, and time-efficient tools such as the Landing Error Scoring System (LESS) represents a crucial initial step [1]. In addition, the predictive value of separate tests is ambiguous, and the current trend is towards the use of test batteries, which approach athletes’ functionality in a more holistic manner [4,10].

The LESS, consisting of 17 separate items, has been suggested to suit this purpose [9,10], as a tool that can be successfully applied during clinical or field-based examinations, with minimal and low-cost equipment [9]. It can also be applied frequently during the season to provide an updated assessment of athletes’ functional status [10]. This is fundamental, because the latter is susceptible to fluctuations during the season, due to injury, illnesses, detraining, and various other factors [4]. In clinical practice, the consideration of the individual scoring items in addition to total scores can provide valuable information about specific deficiencies in landing mechanisms which are crucial for injury prevention [11,12,13]. In addition, combined with other evaluation measures such as strength asymmetries, ballistic function, previous injuries, and workload characteristics, LESS can contribute to the targeted formulation of injury prevention programs and re-evaluate the impact of prevention programs on athletes during the season [10,12]. 

The reliability of LESS depends heavily on examiners paying the appropriate attention to the guidelines while scoring each item. The results of previous studies [9,11,14,15] have indicated good to excellent reliability values of LESS total scores using standard LESS scoring instructions [9]. From these investigations, two reported the inter-rater reliability of the overall instrument, including specific scoring items. Regarding intra-rater reliability, only Everard et al. [15] reported reliability values of a single rater for the individual scoring items except for the total scores. According to these investigations [14,15], despite the range of reliability values of LESS’s specific scoring items, these items provide acceptable reliability. 

The need for additional investigation is dictated by methodological constraints. Earlier research has explored LESS’s reliability in scoring either freshman military subjects or various sports athletes in controlled laboratory settings [14,15]. Expanding the analysis of its reliability in particular groups, such as professional football players or individuals with injuries, would enhance the generalizability of the outcomes [11]. Moreover, it is questionable whether the reliability is similar during in field-based testing, and this has not been tried before. In contrast to evaluations conducted in controlled indoor laboratory settings, outdoor field assessments may be subject to fluctuations in environmental factors and surface conditions, which could impact the assessment of LESS by evaluators. It is also vital to establish reliability among raters with different levels of experience, using solely baseline LESS instructions [9] without further training. In the study by Onate et al. [14], the novice rater had training by the founders of the instrument in the proper scoring technique before the examination of LESS reliability. In case of no requirement for additional training, its use would be expanded.

Therefore, the main aim of the present study was to examine the inter- and intra-rater reliability of LESS in comparing between experienced and novice raters using the standard scoring guidelines proposed by Padua et al. [9]. A secondary aim was to conduct the aforementioned examination on field, since it has been carried out indoors only so far. In this way, this study will provide valuable information for the medical and training staff of football teams for the reliable use of the LESS standard version as part of a team’s injury risk screening protocol, regardless of the venue.

## 2. Materials and Methods

### 2.1. Participants 

Thirty-seven male professional football players from two Greek second-division teams voluntarily participated in this study. The participants’ characteristics are presented in Table 1. The sample of this study was involved in a more extensive prospective cohort investigation examining the risk factors for lower-limb injuries. To assess LESS reliability, participants were chosen randomly from a larger sample. The football athletes involved in this study were free of injury or fully rehabilitated from a previous one, and they participated in their teams’ activities without restrictions. All volunteers were informed of the study’s aim and signed a consent form. Ethical approval was obtained from the Committee of the University of Patras (ID: 12126).

### 2.2. Procedure

During the pre-season period, 37 athletes participated in a field-based drop jump assessment to identify potential risk factors for lower limb injuries. Initially, they were informed of the purpose of the evaluation and signed a consent form. Demographic characteristics, previous season’s training exposure, and injury history were also recorded using a structured questionnaire. Subsequently, after a ten-minute sports-specific warm-up, including running and stretching, athletes performed a bilateral drop-jump task assessment using the LESS guidelines [9].

**Figure 1:**
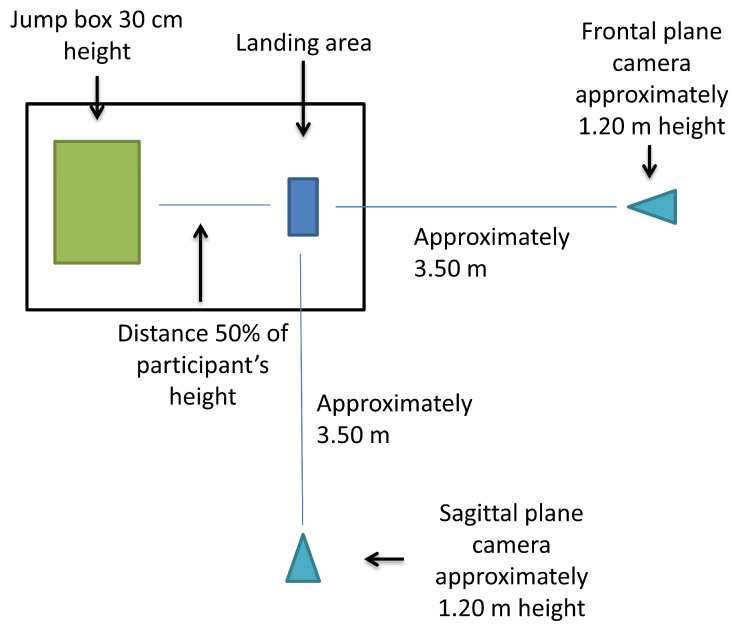
Test setup. Landing area and camera placement.

LESS assessment was conducted in accordance with the instructions provided by Padua et al. [9]. Following the instructions for the appropriate test setup [9], the participants jumped from a 30 cm box at a distance equal to 50% of their height and immediately performed a vertical jump (Figure 1). The athletes were instructed on the proper execution of the task and performed two familiarization trials. Three successful attempts were recorded for analysis. Each athlete spent two to three minutes to complete the test. No instructions regarding the proper landing technique were provided. Two conventional cameras (Sony HDR-CX625 and Panasonic HC-V770) recorded the landing tasks in the sagittal and frontal planes. The recorded videos were stored on the personal computers of two raters for analysis. 

### 2.3. Data Collection

Two raters evaluated the recorded videos of the landings based on published guidelines [9]. To calculate the intra-rater reliability, both raters performed two evaluations of the videos, separated by approximately two months. The videos were evaluated randomly during the second assessment to prevent recall bias. The two raters had different experience levels. The first one had previously used the instructions of Padua et al. [9] when performing approximately 150 LESS evaluations for the purpose of other studies [10,16,17]. The novice rater used the LESS for the first time. Both raters applied the recommended guidelines [9] without further education regarding proper LESS evaluation. The reliability results of the experienced and a novice rater were compared to cover a broad spectrum of raters’ familiarization, since clinical or sports physical therapists usually have little or no respective experience. It is essential to identify the degree to which novice examiners can be trusted when they report LESS values. The experienced rater was a Ph.D. student in sports physical therapy with 10 years of experience in clinical practice, and the novice rater was an M.Sc. student in physical therapy and therapeutic exercise without previous experience in LESS use but with 6 years of experience in clinical practice.

**Figure 2:**
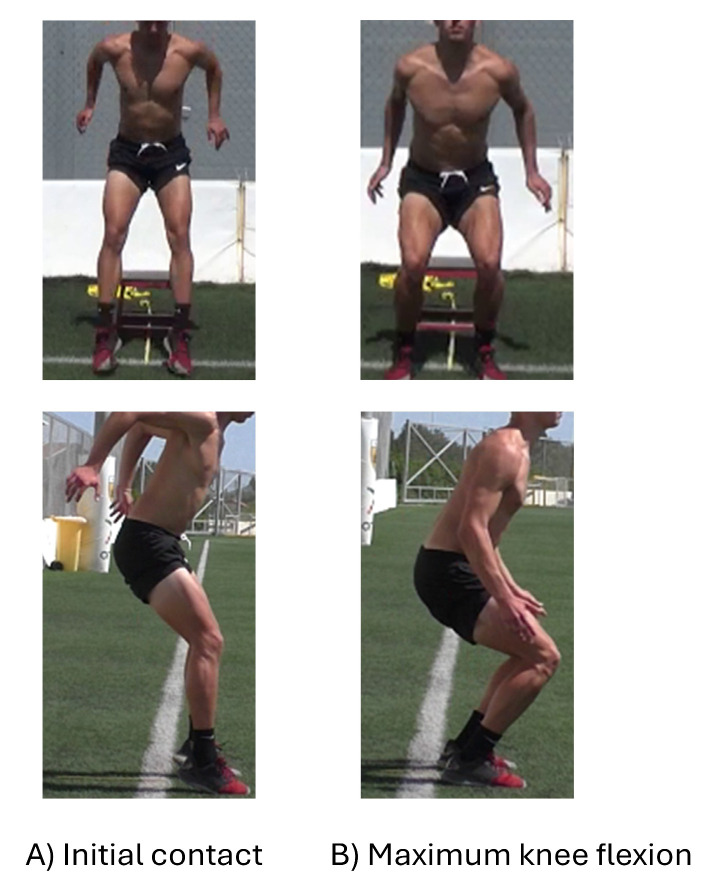
Evaluation of landing mechanism during initial contact (**A**) and maximum knee flexion (**B**) from sagittal and frontal view.

The investigators analyzed the recorded videos of the three bilateral drop-jumps using the scoring sheet that included 17 scoring items [9]. This scoring form focused on the assessment of trunk position, knee (flexion angles, valgus), and hip flexion angle at the initial contact (IC) and the maximum knee flexion during landing (Figure 2). In addition, foot rotation, symmetrical foot initial contact, and base of support width were also considered. Finally, items 16 and 17 reflected the rater’s subjective view of the sagittal plane joint displacement during landing and the overall impression of the landing. For items 1–15, if an error in the landing mechanism existed, the rater scored “1”. For items 16 and 17, the rater scored “0” for soft or excellent landing, “1” for average, and “2” for stiff or poor landing mechanisms. The total LESS score was calculated as the average of the LESS scores of the three trials [12]. Specific errors were recorded if an error existed in at least two of the three trials for items 1–15. For items 16–17, average scoring of “1” is considered when it has been recorded in at least two of the three trials, and for poor/stiff landing scoring, “2” is recorded when it has been recorded in at least in one of three trials. Higher total LESS scores indicate more landing errors. To increase the objectivity of the landing assessment, the videos were evaluated using the two-dimensional video analysis software of Kinovea (Version 0.9.5).

### 2.4. Statistical Analysis 

The SPSS software, version 28, was used for the statistical analysis. The inter- and intra-rater reliability of the total LESS scores were assessed using the average interclass correlation coefficient (ICC) measures for 2-way mixed modes with 95% confidence intervals (CIs). The type of absolute agreement for ICC was used. The kappa statistic was used to evaluate the reliability of the individual scoring items of the LESS, which were categorical/dichotomous (0–1) variables. The kappa statistic has proven to be a more suitable measurement than the percentage agreement [14]. The inter-rater reliability values for the total LESS scores and individual scoring items were calculated for the first and second LESS assessments. For the interpretation of kappa statistics, values of 0 represent poor agreement, 0.01–0.20 represents a slight agreement, 0.21–0.40 represents a fair agreement, 0.41–0.60 moderate agreement, 0.61–0.80 represents substantial agreement, and 0.81–1 almost perfect agreement [18]. Regarding the ICC values, agreement between raters of less than 0.5 indicates weak reliability, 0.5 to 0.75 indicates moderate reliability, 0.75 to 0.90 indicates good reliability, and greater than 0.90 indicates excellent reliability [19]. According to the recommendations of Koo and Li [19], the range of 95% CIs was also considered to provide a more thorough analysis of ICC outcomes.

## 3. Results

The intra-rater reliability of the LESS total scores was excellent for both the experienced and novice raters, with average ICC values of 0.95 (95% CI, 0.89–0.97; *p* < 0.001) and 0.95 (95% CI, 0.90–0.97; *p* < 0.001), respectively. Regarding the inter-rater reliability, the results indicated a good to excellent agreement among raters in the first and second LESS evaluations, with average ICC values of 0.90 (95% CI, 0.77–0.95; *p* < 0.001) and 0.86 (95% CI, 0.71–0.93; *p* < 0.001), respectively. The findings demonstrated a slight variation when examining the range of the 95% confidence intervals. According to the 95% CIs, the results indicated good to excellent intra-rater reliability for both experienced and novice raters. The findings from the inter-rater assessments demonstrated an acceptable level of agreement, with values indicating good to excellent reliability in the initial evaluation and moderate to excellent reliability in the subsequent evaluation. A comprehensive analysis of the descriptive statistics for the raters’ total scores on the LESS scale is provided in Table 2.

The inter-rater agreement of the specific scoring items of the LESS ranged from moderate to almost perfect for most individual scoring items in the first evaluation, and for all scoring items in the second LESS evaluation (Table 3). Low inter-rater agreement was observed in the assessment of the first evaluation for 13 (hip flexion displacement) and 17 (overall impression). However, these specific scoring items reached moderate kappa values when the inter-rater reliability of the second LESS evaluation was assessed. In addition, perfect agreement in both assessments was observed only for items 2 (hip flexion at IC) and 9 (internal foot position). These scoring items presented constant values for all observations. At the same time, we observed a range between substantial and almost perfect agreement when comparing the two evaluations for items 1 (knee flexion at IC), 4 (ankle plantar flexion at IC), 7 (wide stance), 8 (narrow stance), 10 (external foot position), and 12 (knee flexion displacement). Further, there was a moderate inter-rater agreement for item 11 (symmetric foot contact at IC) and a substantial agreement for items 3 (trunk flexion at IC) and 15 (knee valgus displacement) in both evaluations. Finally, we observed moderate to substantial agreement for items 5 (knee valgus at IC), 6 (lateral trunk flexion at IC), 14 (trunk flexion displacement), and 16 (overall joint displacement) when we compared the two evaluations.

The intra-rater values of the experienced and novice raters ranged from moderate to almost perfect agreement (Table 3). Almost perfect intra-rater reliability for both raters was observed for items 2 (hip flexion at IC), 3 (trunk flexion at IC), 4 (ankle plantar flexion at IC), 9 (internal foot position), and 14 (trunk flexion displacement). In addition, raters achieved substantial intra-rater kappa values for items 11 (symmetric foot contact at the IC) and 13 (hip flexion displacement). The remaining items had substantial and almost perfect intra-rater agreement for both raters. Finally, there was moderate intra-rater agreement for both raters on item 17 regarding the overall impression of the landing technique, representing the lowest intra-rater values for both raters.

A final outcome revealed that determining an athlete’s performance using the LESS necessitates diverse scoring durations among evaluators. A novice rater typically takes up to 20 min, while experienced rater spends an average of 10 min.

## 4. Discussion

The current research examined the reliability of LESS in and between raters with varying levels of experience in its application. Further, this study was conducted on field during pre-season screening of professional football players. The outcomes of our research showed that raters with varying backgrounds in LESS evaluation and without further training achieved excellent intra-rater reliability and very good to excellent inter-rater agreement in LESS total scores. These findings align with previous studies [14,15], which reported similar ICC values for LESS total scores. In addition, our study provides inter- and intra-rater reliability values for the individual scoring items. To the best of our knowledge, this is the first study to examine the reliability of LESS among professional football players in field-based situations. We chose to investigate the intra- and inter-rater reliability among raters with no previous training in adequately using the LESS instrument and following only the standard instruction and scoring sheets of Padua et al. [9]. Our purpose was to assess whether the LESS has the appropriate reliability to be adequately used by the football team’s medical staff, using only the instructions given by the founders of the instrument as a guide [9]. 

According to the results, the LESS total scores had excellent intra-rater and very good to excellent inter-rater reliability values. These results agree with previous studies that examined the reliability of the LESS total scores in various sports athletes under laboratory conditions [11,14,15]. The total scores provide an overall impression of the landing technique, with lower values indicating a safer landing mechanism, with a lower risk of injury [9,12]. Athletes who score over five [12] or six [13] are categorized as having high-risk landing performance. Using these cutoff points, the team’s medical and coaching staff can classify athletes as high- or low-risk for injury in combination with holistic field-based screening measurements, including isometric strength parameters, triple-hop landing, flexibility, and core stability measurements [10]. Subsequently, the team’s staff can implement appropriate injury-prevention exercise programs for at-risk athletes [2,10,11]. However, the total LESS score results may be similar on different occasions despite differences in the scores of individual scoring items [15].

The individual scoring items can provide insight into specific deficits of landing. In contrast to the good to excellent reliability values of the LESS total scores, the reliability values of individual scoring items should be considered more cautiously. Our findings indicate a degree of inconsistency in the reliability scores of the individual scoring items. Specifically, the results of some individual items, such as knee flexion at IC, hip flexion displacement, and wide stance, revealed a certain degree of variability in the inter-rater reliability values across the first and second evaluations. Furthermore, the overall impression (item 17) demonstrated low reliability values. Consequently, sports physical therapists must thoughtfully assess the outcomes of these specific items when assessing for possible risk factors or the efficacy of preventive measures based on individual LESS items. However, in agreement with previous studies [14,15], the inter- and intra-rater reliability for most of the scoring items in our research ranged from moderate to almost perfect agreement. 

It is important to mention differences between our study and previous investigations, because these may affect the comparison of the results. Particularly, the study of Onate et al. [14] used only the first trial to calculate inter-rater reliability values and not the average results of the three trials proposed by Padua et al. [9]. On the other hand, the study of Everard et al. [15] reported that while raters had no experience using the LESS, they had significant experience with the Functional Movement Screen (FMS), which may have affected the results, as the authors mentioned in their paper. Finally, as Everard et al. [15] noted, the influence of rater background on LESS results requires further examination.

Specifically, regarding the inter-rater reliability values of individual scoring items, substantial and moderate agreement in the repeated evaluation of the videos was observed for most scoring items, which agrees with previous studies [14,15]. Nevertheless, in contrast with the relevant literature [14,15], low inter-rater kappa values (<0.40) were observed in the first evaluation of the videos regarding the hip flexion displacement between the IC and peak knee flexion (item 13). However, Everard et al. [15] reported an inter-rater agreement of 0.53, which was slightly different from the inter-rater agreement of item 13 (0.36) in this study. At the same time, lower inter-rater values than those reported in the literature [15] were observed in the evaluation of item 17 and, more precisely, in the first assessment. This can be attributed to the scoring of the overall impression of the landing technique (item 17), which is strongly subjective, despite instructions, and it is influenced by the level of the rater’s experience as well as their specific training on the LESS or other relative tests such as the FMS [14,15]. However, we observed moderate kappa values for the 13 and 17 scoring items in the second inter-rater evaluation. As previously mentioned, our study raters had different experiences using LESS, but neither had received education about the proper technique of rating, which may have influenced the low inter-rater reliability values of items 13 and, more importantly, item 17. 

In clinical practice, different clinicians may assess the landing technique of an individual before and after the implementation of injury prevention programs. Considering the subjective nature of individual scoring of some items, we calculated the inter-rater reliability of scoring items at two different time points to evaluate the stability of the reliability results. The results indicated that the inter-rater reliability values of most scoring items ranged between the two video assessments. A possible explanation for this phenomenon is that, because the scoring follows a dichotomous strategy (score 0 or 1) when a landing impairment is near the cutoff point, the score given by a rater may change at different scoring times. For instance, this may explain the low inter-rater reliability values for hip flexion displacement (item 13). Despite these variations in values, the scoring form seems to have an acceptable inter-rater agreement, and clinicians can be informed about the possible specific landing impairments of their athletes. In addition, clinicians should be aware that because of the dichotomous process of scoring each impairment, observation is not very sensitive. For instance, whereas we rated the existence of valgus during landing, the instrument does not inform us about the magnitude of the valgus presented in a participant. These observations can be added to the total score only when the rater scores item 17 regarding the overall impression of landing, which is clearly a subjective scoring criterion, as we have already mentioned.

To our knowledge, there is limited evidence in the literature regarding the intra-rater reliability of two raters with different experiences using the LESS instrument. Onate et al. [14] examined only the inter-rater agreement among an expert and novice rater, whereas the study of Everard et al. [15] provides intra-rater values only from the one rater. Similar to Everard et al. [15], our results indicate that the kappa values showed substantial and almost perfect agreement for most scoring items. Furthermore, both raters showed moderate agreement in overall landing impressions and substantial agreement in general joint displacement. 

In addition, a side finding of the study revealed that the two raters differed in the time required to complete the scoring. Specifically, the novice rater spent up to 20 min rating each participant’s three attempts, whereas the experienced rater spent 10 min on average. This may be because the novice rater usually spent more time scrutinizing and reading the scoring instructions. The control of scoring time, in future studies, will provide valuable information about the aforementioned observation. Finally, future attempts could add a specifically trained rater in LESS.

In conclusion, sports physical therapists can reliably use the LESS total scores to assess the landing technique of football players, following only the published instructions of the standard version of the instrument [9]. LESS is a valuable tool for medical teams, regardless of the examiners’ level of experience, for field-based screening of football players. However, the individual scoring items should be viewed with caution since there was a considerable range in reliability. In any case, information of this assessment tool can draw attention to specific landing impairments of each individual, and along with additional testing, an individualized exercise prevention program can be planned. However, its direct connection to injuries remains to be further investigated.

## Figures and Tables

**Table 1:** Samples’ demographic characteristics.

	Minimum	Maximum	Mean ± SD
Age	17	32	21.49 ± 4.06
Weight (kg)	63.68	93.13	74.92 ± 7.25
Height (m)	1.66	1.92	1.81 ± 0.07
BMI	20.47	25.77	22.92 ± 1.37

**Table 2:** Descriptive statistics of LESS total scores.

	Minimum	Maximum	Mean ± SD
Experience 1	1.67	11	5.65 ± 1.88
Experience 2	2.67	10	5.95 ± 1.69
Novice 1	1.67	10.67	5.12 ± 1.89
Novice 2	2.33	11	5.4 ± 2.1

**Table 3:** Inter- and intra-rater agreement of specific scoring items of the experienced and novice raters.

	Scoring Items	Inter-Rater Cohen’s Kappa First Evaluation *	Inter-Rater Cohen’s Kappa Second Evaluation *	Intra-Rater Cohen’s Kappa Experienced Rater *	Intra-Rater Cohen’s Kappa Novice Rater *
1	Knee flexion at IC	0.92	0.62	0.71	1.00
2	Hip flexion at IC	1.00 (constant)	1.00 (constant)	1.00 (constant)	1.00 (constant)
3	Trunk flexion at IC	0.65	0.64	1.00	1.00
4	Ankle plantar flexion at IC	0.80	0.87	0.93	1.00
5	Knee valgus at IC	0.59	0.68	0.80	0.65
6	Lateral trunk flexion at IC	0.72	0.53	0.65	0.80
7	Wide stance	0.65	1.00	0.65	1.00
8	Narrow stance	0.89	0.74	0.79	0.95
9	Internal foot position	1.00 (constant)	1.00 (constant)	1.00 (constant)	1.00 (constant)
10	External foot position	0.92	0.71	0.77	0.85
11	Symmetric foot contact at IC	0.60	0.60	0.80	0.80
12	Knee flexion displacement	1.00	0.68	0.68	1.00
13	Hip flexion displacement	0.36	0.53	0.64	0.64
14	Trunk flexion displacement	0.54	0.63	0.94	0.83
15	Knee valgus displacement	0.63	0.72	0.68	0.89
16	Overall joint displacement	0.66	0.62	0.71	0.83
17	Overall impression	0.20 **	0.44	0.51	0.43

* Statistically significant < 0.05; ** not statistically significant = 0.12; constant—all rates have the same value.

## Data Availability

The data presented in this study are available on request from the corresponding author. The data are not publicly available due to privacy and ethical restrictions.
